# Ethical challenges for medical professionals in middle manager positions: a debate article

**DOI:** 10.1186/s13037-015-0073-6

**Published:** 2015-06-30

**Authors:** Joerg Schnoor, Christoph-Eckhard Heyde, Mohamed Ghanem

**Affiliations:** Department of Anesthesia and Intensive Care Medicine, University Hospital Leipzig, Liebigstraße 20, Leipzig, 04103 Germany; Department of Orthopedics, Traumatology and Plastic Surgery, University Hospital Leipzig, Liebigstraße 20, Leipzig, 04103 Germany

**Keywords:** Middle manager, Medical professionals, Ethical challenge, Economy

## Abstract

**Background:**

Demographic changes increase the financing needs of all social services. This change also generates new and complex demands on the medical staff. Accordingly, medical professionals in middle management positions hold a characteristic sandwich position between top management and the operational core. This sandwich position often constitutes new challenges. In the industrial field, the growing importance of the middle management for the company’s success has already been recognized. Accordingly, the growing demand on economy urges an analysis for the medical field.

**Discussion:**

While there are nearly no differences in the nature of the tasks of medical middle manager in the areas of strategy, role function, performance pressure and qualifications compared to those tasks of the industrial sector, there are basic differences as well. Especially the character of “independence” of the medical profession and its ethical values justifies these differences. Consequently, qualification of medical professionals may not be solely based on medical academic career. It is also based on the personal ability or potential to lead and to manage.

**Summary:**

Above all, the character of “independence” of the medical profession and its ethical values justifies medical action that is based on the patient’s well-being and not exclusively on economic outcomes. In the future, medical middle managers are supposed to achieve an optimized balance between a patient-centered medicine and economic measures. It will be a basic requirement that middle managers accept their position and the resultant tasks putting themselves in a more active position. Because of that, middle managers can become “value-added bridge-builders”.

## Background

Industrialized countries are affected by demographic development, which increases the financing needs of all social services. Consequently, new and complex demands are put on the medical staff. On one hand, a change from paternalism takes place to a shared decision-making that highlights a targeted promotion of patient autonomy. On the other hand, an underfunding of the health care system induces physicians’ development to a guardian of limited resources, which might increasingly threaten the historical basis for confident and sustainable doctor-patient relationship.

Similar to the industrial sector, medical professionals in middle manager positions holds such a characteristic sandwich position between top management and the operational core. Especially for ambitious employees a middle manager position seems to be both attractive and an unavoidable step-over place on their way to greater responsibility. With it, they have to face new challenges [1–3].

In the industry, the growing importance of middle management for the company’s success has been recognized several decades ago [1, 2, 4]. For the medical field this discussion, however, remained largely ignored, although cooperation and alignment between medical staff and hospitals has become paramount to enhance hospital performance [5, 6]. The growing demand on the economy, however, urges an analysis, precisely because - in contrast to the industry - the middle manager in medicine occupies a special position in which ethical principles make the difference.

Therefore, this manuscript here should allow a closer examination of the problem. We’ll try to delineate the typical characteristics of industrial middle managers prior to discussing the functions and needs of middle managers in the medical sector with its unique features including future options for solutions.

## Discussion

### Characteristics of industrial middle managers

Due to the varied requirements the definition of middle management should take into account the objectives of each company [4]. In business, middle management acts as a link between the strategic apex and the operating core. Middle managers simultaneously perform managerial as well as specialized specific tasks. Typical challenges that face middle managers can be outlined in five areas: strategy, role function, performance pressure, skills and ethics [2]:

#### Strategy

The overall strategy of middle management in a company is to take over the tasks of transfer and transformation of information of the overall strategy into practical sub-strategies. The middle management translates corporate strategy into operational sub-steps and serves as an information broker to the operating units. In the ideal case, the middle manager also transfers the information from the operating core to the top management and thereby creates the information base for strategic decisions of top management. The technical and management expertise of middle managers enables an implementation of strategies into concrete action. In this context, specific problem areas can result (Table 1).

**Table 1:** Areas of conflict for middle managers (adapted to [2])

	Areas of conflict for middle managers (MM)
Strategies	• MM are usually not involved in strategic considerations [6]. • A limited freedom of creative leeway can demotivate and paralyze [6]. • Imprecise / unrealistically framed strategies require “silent” adaptations (emergent strategies) [7]. • Achievements are usually awarded to the top management [2]. • Failure of a strategy or failure of implementation of strategy will be attributed to the MM [2].
Role function	• MM feel an increasing pressure to succeed with decreasing appreciation of their input [24]. • Compared with the top management, MM try to defend their freedom of action and choice [25]. • Compared to its own employees, MM try to assert their own position [25]. • Various loyalty claims generate stress fields with ambivalence and role dissonance. • The interface position requires the fulfilment of the “trouble shooter and scapegoat” [26]. • Restructuring/rationalization is carried out to the detriment of the MM (job-, image loss) [27, 28].
Pressure to perform	• MM should resolve conflicts and motivate and create trust [25]. • Authenticity, leadership and management responsibilities are expected from MM. • The diversity of loyalty towards different stakeholders creates ambivalences [29]. • The commitment to loyalty to multiple senior staff members can generate the allegation of a lack of loyalty to one or more.
Qualification	• For MM time was too short to prepare themselves for new requirements. • The advancement from a colleague to a supervisor can be perceived as problematic [30]. • Personnel management measures put high demands on communication and conflict skills. • Young managers need to develop their own leadership style. • Unclear instructions have to be transferred into clear measures by the MM. • An obstacle of delegation can overwhelm the MM, while employees might be under-challenged.
Ethics	• The acquisition of ethical values of the company may require the ignorance of own values. • Exceeding moral limits impose frustrations, uncertainties, and loss of authority. • A loss of ethical values within a company can demotivate. • When ethical conflicts become publicized, MM is rapidly becoming a “pawn”. Job losses and the loss of reputation as well as a loss of self-esteem may then assume health-threatening proportions.

#### Role function

The middle manager takes on different roles within the individual levels of the enterprise, which may contradict each other. Middle managers are simultaneously supervisors and employees. They act as a receiver and transmitter of information, orders and arrangements. As part of their management and control functions middle managers run both employees and production or service processes. Thus, they exert a direct influence on the allocation of resources. Middle managers also act as a motivator and communicator. They are knowledge providers with a particular focus on the transfer of mission-critical knowledge. In addition, the middle managers should act in the interest of top management, the own employees, external customers and the cooperating partners. This might results in areas of conflict (Table 1).

#### Pressure to perform

Pressure to perform is considered to be a specific problem of the middle manager, which is caused by the “sandwich” position with growing complexity of the task. The mechanization and globalization increase the need for coordinating working processes. Similarly, the growing complexity of the tasks of the middle manager makes their role more complex. Middle managers should take the needs and wishes of employees, customers, partners and suppliers into account. Facing the resistances of individual actors the middle manager should provide acceptable compromises in the interests of the company. Therefore, middle managers act as a link between the different interest groups (Table 1).

#### Qualification

The necessary qualifications of the middle manager go beyond the actual technical expertise. The middle manager is often generated out of the employees of the operating core. For this purpose, additional qualifications in the field of personnel management are required (Table 1).

#### Ethics

The ethical understanding of a company is rooted in the corporate culture. It consists first of general principles. What is considered “good”, however, depends on the particular circumstances. An economic calculus in this case provides room to relativize the general principles of “good” action in order to achieve the “higher” objective: profit maximization. The industrial middle manager constitutes the intersecting point of different fields of interests and different hierarchical levels. Hence, the middle manager should sometimes implement decisions of which he is not convinced (Table 1).

### From the industry to medicine

In general, each organization with integrity, which is playing an active role in aligning ethical and economical values, will be able to systematically address the competing interests of patient care and profitability. Meanwhile, multimorbidity and medical advances generate a growing challenge to the financing strategies of health care. Hospitals are increasingly forced to pay off their investment from the treatment proceeds. For this purpose, the number of cases was increased. This resulted in a transformation of the historical state precautionary principle into a healthcare market with tough competition [7, 8]. At the same time, a paradigm shift has taken place. With the growing demand for patient autonomy, safety and quality of results in terms of a demand for improved quality of life, the change can be from paternalism towards the “ shared decision making ” [9, 10]. Germany responded defensively on patient autonomy and enforceable medical services with a “legalization and standardization” of a “medicine of the over-diagnosis” [11]. This attitude triggers more costs and hence implicates increased need of medical actors to meet economic targets.

### The middle manager in medicine

**Fig. 1:**
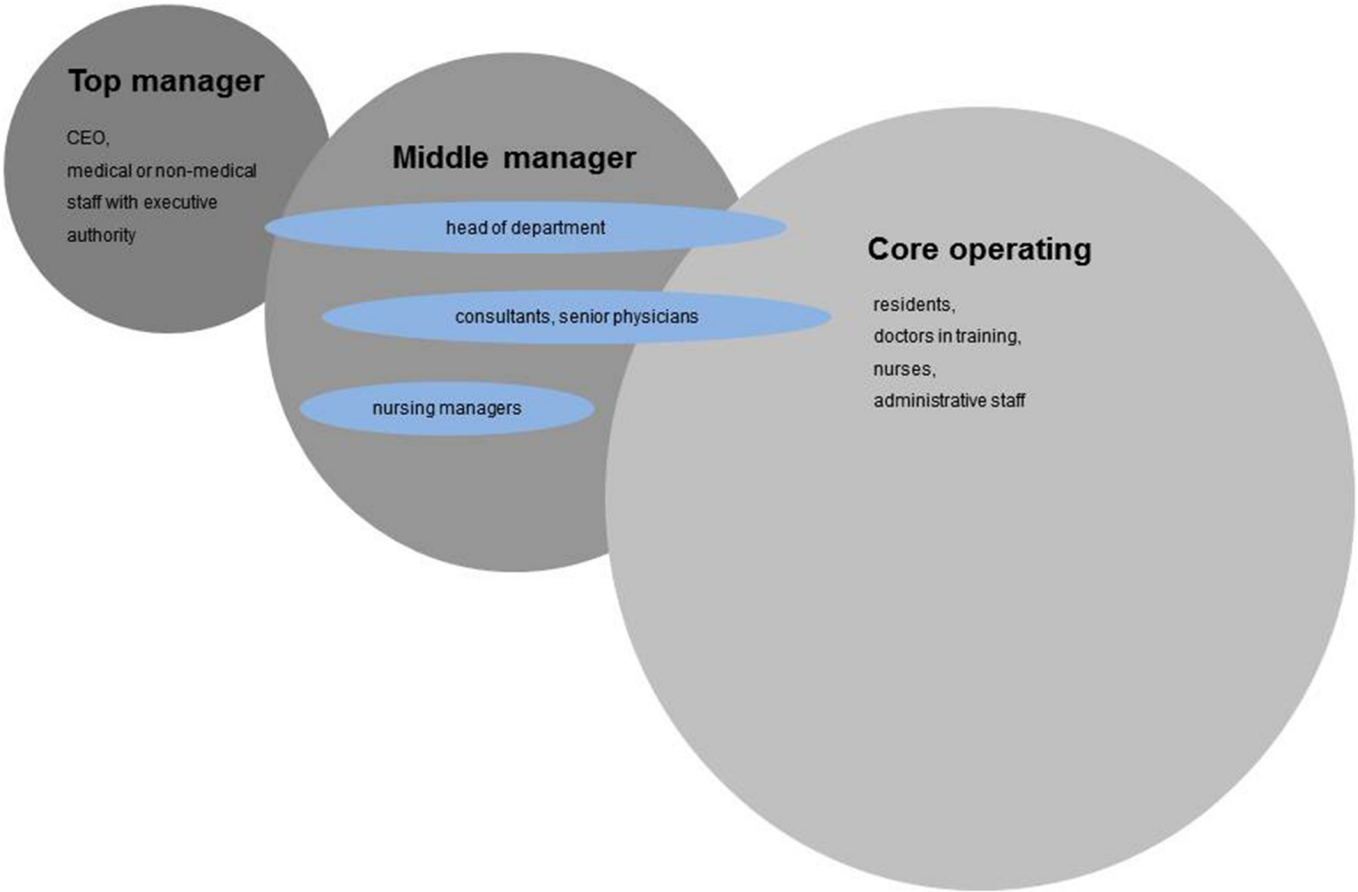
Sandwich-position of the middle managers in medicine

The company’s organizational structure also influences the respective functions of the medical middle manager. In our opinion, the middle manager in a hospital can be defined as follows: First, top management includes CEO and medical and/or non- medical staff with executive power (e.g., medical director with the management responsibility for several hospitals). Middle management is basically formed by heads of departments or directors of services. In addition, this task can also be acquired by practice providers, consultants, nursing managers, ward and department nursing managers. The operating core consists of specialists, residents, doctors in training, the nursing staff as well as administrative employees without managerial function (Fig. 1).

In this manner, employees might also take overlapping functions. Chief physicians with activities in patient care act in the operating core and might simultaneously practice top management functions through comprehensive management tasks. In the following middle management will be considered on the example of heads of departments and consultants.

#### Medico-ethically justified differences compared to the industry

While we found nearly no differences in the nature of the tasks of medical middle manager in the areas of strategy, role function, performance pressure and qualifications compared to those tasks of the industrial sector, obviously, there are basic differences in the ethical field. These differences are justified especially by the character of “independence” of the medical profession and its ethical values.

#### Professional code of the independent medical profession

The pattern of professional code of the German Medical Syndicate lists the ethical rules of the medical profession [12]. Accordingly, the medical activities of the physician belong to the independent professions. According to their conscience, physicians shall act in the way that is dictated by medical ethics and humanity. Here, the medical profession is conscientious and is exercised for the benefit of patients. In particular, physicians may not represent the interest of third parties on the cost of the patient well-being or take instructions from non- physicians regarding their medical decisions.

This ethical and moral set of values also characterizes the function of the medical middle manager. Contrary to the industrial field, the primary obligation is to protect and enhance the patients’ well-being [13, 14]. Therefore, the free directive authority of the middle manager concerning medical decisions outweighs the organizational managerial hierarchical structure related to its status as employee. Although the employer enjoys a general right to give instructions, e.g., determine the individual working conditions, the free medical directive authority remains the all-important defining feature of the medical profession. Thus, hospital top management authority can determine strategy as long as these orders do not interfere with the professional medical activity on the patient [15].

Regarding the medical profession, senior medical staff members hold directive authority. However, the independent medical responsibility also limits this authority due to the fact that tasks are assigned by senior staff to younger staff members, who take over the direct responsibility of what they perform on the patient [15]. This makes medical middle managers again not bound by instructions in the framework of its activities on the patient. Medical reasons can even force a contradiction to orders of top management, since a breach of the obligation to provide care to patients could lead to a withdrawal of the license to practice medicine according to which the middle manager is threatened by a prohibition.

In contrast, the industrial middle managers are obliged to follow corporate objectives. He is bound by instructions as a result of a directive authority of the employer. If the middle manager professionally or morally disagrees, this would be regarded as ignoring an order. The threatened consequence would be its exemption. Yet, he is not threatened by prohibition of employment.

#### From the process decomposition to attribution of failure

In addition to the ethics-based differences, German medical managers increasingly have to face a rising coordination effort of previously decomposed process chains [16]. Their successful (re-)coordination requires precise knowledge of the process, a continuous presence and corresponding decision-making power. This allows the middle manager to adapt orders to clinical reality, so that a project can successfully be performed (emergent strategy) [17]. A failure would otherwise be attributed to the middle manager (attribution of failure [2]). Serious conflicts of loyalty often arise due to the complex interface links for the middle managers. If conflicts of interest stay ignored and are passed forwards by middle manager on a top-down basis, the operating core would suffer a conscience crisis between the targets and the ethical principles. Sooner or later, this means the middle manager will either miss its target or lose the support of the operating core.

The resulting basic problems of medical middle management as a doer with great responsibility but limited decision-making and creative power can be summarized as follows:

1. The top priority of the medical middle manager is considering his professional ethics.
2. At the same time the middle manager must be loyal to his employer.
3. The commitment increasingly implicates profit-oriented performance.
4. Consequently, the middle manager can get into existential conscience crisis.

### Solutions

Being primarily committed to the professional code of conduct, the medical middle manager can still take over the function of a value-adding bridge builder. For this he needed a clear position in the field of action between economics and patient well-being.

#### Tools of industry

Industrial solutions are based on a management culture of mutual recognition and appreciation. Values of fairness, cooperation and trust are awarded to an “honest businessman”. This spirit should be exemplified by top management in a top-down way. The middle management serves as a promoter. Middle managers are introduced to their tasks through targeted employee-oriented human resources and organizational development. Depending on the structure and objectives of the company, individual development of management skills are carried out to ensure dealing with coordination, design and development tasks [1]. Table 2 demonstrates the conclusions of the expert Delphi [2]:

**Table 2:** Solutions for industrial middle managers

	Solutions for middle managers (MM)
Strategies	• Involvement and participation in the strategic development • Correction of unrealistic and incorrect strategies
Role definition	• Reflection on the role along with top management (clear mission, expectations and goals) • Precise definition of the area of responsibility • Clear action skills • Clear instructions for conflict cases • Definition of career objective “middle management” for sustainable business development • Move away from the image of the transit station on the career ladder • Demand and promoting a sense of responsibility and authenticity
Pressure to perform	• Free space for balance between autonomous and heteronymous leadership • Deals for professional accompanying consultation, mentoring and coaching • Offers for communication and conflict training • Flexible working hours in the interest of MM • Offers for health promotion in the interest of MM
Qualification	• Professionalization of management of operational communication processes • Targeted training concepts for operational change management • Programs aiming at achieving advancement in the company (horizontal career paths)
Incentives	• Salary levels in relation to top management and the operational core • Time quotas for qualifications and further training • Time quotas for self-reflection • Flattening of hierarchies
Ethics	• Formulation and anchorage of a corporate code • Code of Conduct training for all new hires • Antecedents of the company values at all levels, in particular by the top management • None (not-lived) Ethics formulation for marketing reasons • Active and open communication culture, possibly any specific communication channels for ethical issues • Regular employee surveys

#### Tool for the medical

The extent to which the more general recommendations concerning accomplishing goals of enterprises are applicable in the medical sector remains open. The various offers of numerous consultancies for medical leadership seminars in the medical sector also point to a growing activism. However, the specific characteristics of the health service require its own management skills. Providers of management seminars can rarely show a clinical expertise, so that the development of leadership techniques alone are hardly useful for the real hospital operating needs. The renaissance of old virtues (authenticity, honesty, respect, etc.) is not only taught to managers through Flowcharts or in role-play. In medicine, it is necessary for this purpose to ensure high personal maturation together with a team-oriented behavior, meanwhile placing patients’ well-being as top priority. This can certainly be embedded in training and mentoring program. The aspired personal maturity in leadership requires an individual development time. In the DRG era, time is a rare commodity that is hard to get. This is attributed to the fact executives tend to minimize personnel costs, a method which still remains one of the few measures to reduce costs and to allow for profit. The current DRG system thus contradicts any investment in time. In contrast, the system promotes the strict focus on the company’s balance sheet. Thus, the funding system will sooner continue to hinder any sustainable investment. In this context, the development of appropriate management cultures remains subject to the constraints of the payment system.

Trust requires robust arrangements in a defined framework. To develop a hierarchical structure in a collegial active organization, a culture of “360°-feedback” is needed in which constructive criticism is encouraged. Original solution concepts would be desirable from the hands of clinicians who are familiar with the ethical implications of economic decisions on the patient. This ensures sustainable management culture development. In future, medicine should learn to redefine the role of the doctor especially concerning the distinction between therapy and profitability, especially to counter the growing threat to freedom and independency of treatment which resulted due to the increasing influence of economics on the doctor-patient relationship [18–20]. Therefore, it should be considered whether medical students should be prepared more intensively in order to cope with these challenges. This might contain teaching basic rules of medical profession, ethics and economic principles in order to be better prepared for the practical decisions in daily life as well as in problematic situations within the team. Overall, the inherent contradictions of the health care financing system can only be recognized with both knowledge of the lump-sum payment system itself and the resulting medico-legal requirements. This knowledge helps in finding the right decisions to meet patients need, particularly in critical situations.

The medical work is and will remain a service to people on the basis of an oriented care and sound doctor-patient relationship. The team performance character in medicine enforces the aspiration for an overall solution for all concerned. Not only the chief physicians, but also especially the cooperating professional organizations and professional associations could provide a useful interdisciplinary consensus, including backing up those concerned about their limits within their own sector boundaries. Basically, however, a systematic solution to the problem of remuneration will be needed, which ultimately implicates involvement of the responsible policy makers [20].

In this context, priority should be given to increasing the number of managers that possess both the clinical and the managerial skills. Further, it has been already emphasized that hospitals should work on getting all employees to speak a common language paving the way for understanding and accepting each other’s opinion [21, 22]. Accordingly, courses, workshops or even par time graduate postgraduate education in business and economics is recommended for the medical staff. As far as nonmedical hospital executives are concerned, a study recommended hosting them in a hospital department for a period of 6–12 months, introducing them to the daily medical activities, of course without self-performing sophisticated medical care. The supervising medical staff might certify this. Such a certificate and training program can be considered as a prerequisite for occupying top management activities [23].

In medicine, qualification should not be solely based on medical academic career. It might be also based on the personal ability or potential to lead and to manage in addition to the experience on the pure medical field. The ability to lead and manage can be enhanced and developed by complementary education in business, economics and leadership.

## Summary

Medical professionals in middle management positions hold a characteristic sandwich position between top management and the operational core. While there are nearly no differences in the nature of the tasks of medical middle manager in the areas of strategy, role function, performance pressure and qualifications compared to those tasks of the industrial sector, there are basic differences in ethical aspects. Above all, the character of “independence” of the medical profession and its ethical values justifies medical action that is based on the patient’s well-being, whilst the economic outcome still remains an important but subordinated goal. In the future, medical middle managers are supposed to achieve the optimized balance between a patient-centered medicine and economic measures. It will be a basic requirement that middle managers accept their position and the resultant tasks putting themselves in an active position. Because of that, middle managers can become “value-added bridge-builders”.

## Abbreviation

DRG: Diagnosis-Related Groups
